# Sweet Syndrome With Vasculitis: Time To Adopt a New Criteria?

**DOI:** 10.7759/cureus.48399

**Published:** 2023-11-06

**Authors:** Muhammad Hassan Shakir, Salman A Basit, Syed Muhammad Hussain Zaidi, Sarasija Natarajan, Omar Z Syed, Mohammad Asim Amjad, Douglas Klamp

**Affiliations:** 1 Internal Medicine, The Wright Center for Graduate Medical Education, Scranton, USA; 2 Internal Medicine, Shifa Tameer-E-Millat University Shifa College of Medicine, Islamabad, PAK

**Keywords:** national organization for rare disorders, robert douglas sweet, gomm-button disease, cutaneous papules, acute febrile neutrophilic dermatosis, neutrophilic dermatosis, diagnostic criteria, vasculitis, sweet syndrome

## Abstract

Sweet syndrome (SS) is an acute febrile neutrophilic dermatosis. Although perceived to be rare, the disease may well have been underreported due to lack of exposure in low-volume clinical settings and due to the use of rather strict clinical criteria for diagnosis. It presents as cutaneous papules, plaques, or nodules in an asymmetric distribution that follows fever and flu-like symptoms. Data on the disease is ever-expanding. Several associations have been identified, including drugs, infections, malignancies, and autoimmune diseases. Different disease patterns and histological variants have been identified. Pathophysiology is complex and multifactorial but appears to involve mechanisms that negatively influence neutrophil apoptosis and facilitate neutrophil recruitment. The existing diagnostic criteria exclude cases with vasculitis; over time, cases of neutrophilic dermatoses with vasculitis have been reported as SS as long as other criteria were met. Newer diagnostic models have been proposed, some arguing against the exclusion of vasculitis. Steroids continue to be the mainstay of treatment, and steroid responsiveness continues to be a part of the diagnostic criteria, although newer treatment modalities have been used and have shown promise. No established guidelines exist for management. We present a case of Idiopathic SS with vasculitis along with a brief review of the existing literature. We agree to the inclusion of vasculitis as proposed by the newer diagnostic criteria.

## Introduction

Sweet syndrome (SS) is an acute febrile neutrophilic dermatosis without vasculitis that was first described by Dr. Robert Douglas Sweet in 1964 [1]. It was initially referred to as “Gomm-Button disease” in honor of the first two patients. All of his first eight patients were females between the ages of 32 and 55 years.

The typical presentation involves the acute onset of tender, erythematous plaques/nodules of varying sizes in an asymmetrical distribution. Fever and arthralgias are commonly present. The disease is rare with a female-to-male predilection of 4:1 [2]. The age of presentation ranges between 30 and 60 years, although cases have also been reported in age extremes. As per the National Organization for Rare Disorders report on SS from 2015, several hundred cases have been reported, with almost 80 cases in children. Data on the etiology, pathophysiology, clinical presentation, and histological variants is ever expanding. We report a case of SS with atypical histological findings.

## Case presentation

A 79-year-old female, with a past medical history of mitral and aortic valve replacement (on warfarin, not on any immunosuppressive medications), hyperlipidemia, chronic obstructive pulmonary disease (COPD), and a 40-pack-year smoking history (quit five years prior), presented to the emergency room with a new onset skin rash for one day. She did not report any joint pains, fever, fatigue, oral ulcers, chest pain, shortness of breath, epistaxis, or any other upper respiratory symptoms. She did not report any prodromal symptoms. The rash involved bilateral palms, thighs, and knees. The lesions were painful and enlarging since onset. She also reported subjective chills. Her family also reported her being confused and irritable since the onset of symptoms. She denied sick contacts. She denied any new pets. There was no history of recent travel outside the state. She did not report noticing any tick bites. She denied the use of any new medications except for diclofenac, which was prescribed for a recent shoulder injury. She did not have any known drug allergies. On examination, she was afebrile, hemodynamically stable, and alert, but pleasantly confused. Examination showed multiple raised, tender, bluish-purple, deep-seated, nonblanching papular lesions involving palms, soles, and anterior thighs bilaterally. The lesions were ranging from 3 mm to 1 cm in maximum diameter. The examination was otherwise unremarkable. Admission labs were significant for white blood cell (WBC) count of 20k, erythrocyte sedimentation rate (ESR) 68 mm/hour, and C-reactive protein (CRP) 187 mh/dl. The metabolic panel was within normal limits. Blood cultures were drawn and she was empirically treated with ceftriaxone, doxycycline, and vancomycin.

Over the next few days, the patient had multiple fever spikes with T_max_ of 39.6 C/103.3 F. A viral hepatitis panel and tickborne disease panel (including *Ehrlichia*, *Anaplasma*, Lyme disease panel, and Ricketsia) were negative. Serum rapid plasma reagin (RPR) was reactive although the fluorescent treponemal antibody absorption test (FTA-ABS) was negative. Transesophageal echocardiography ruled out valvular vegetation. Blood cultures continued to be negative.

**Figure 1:**
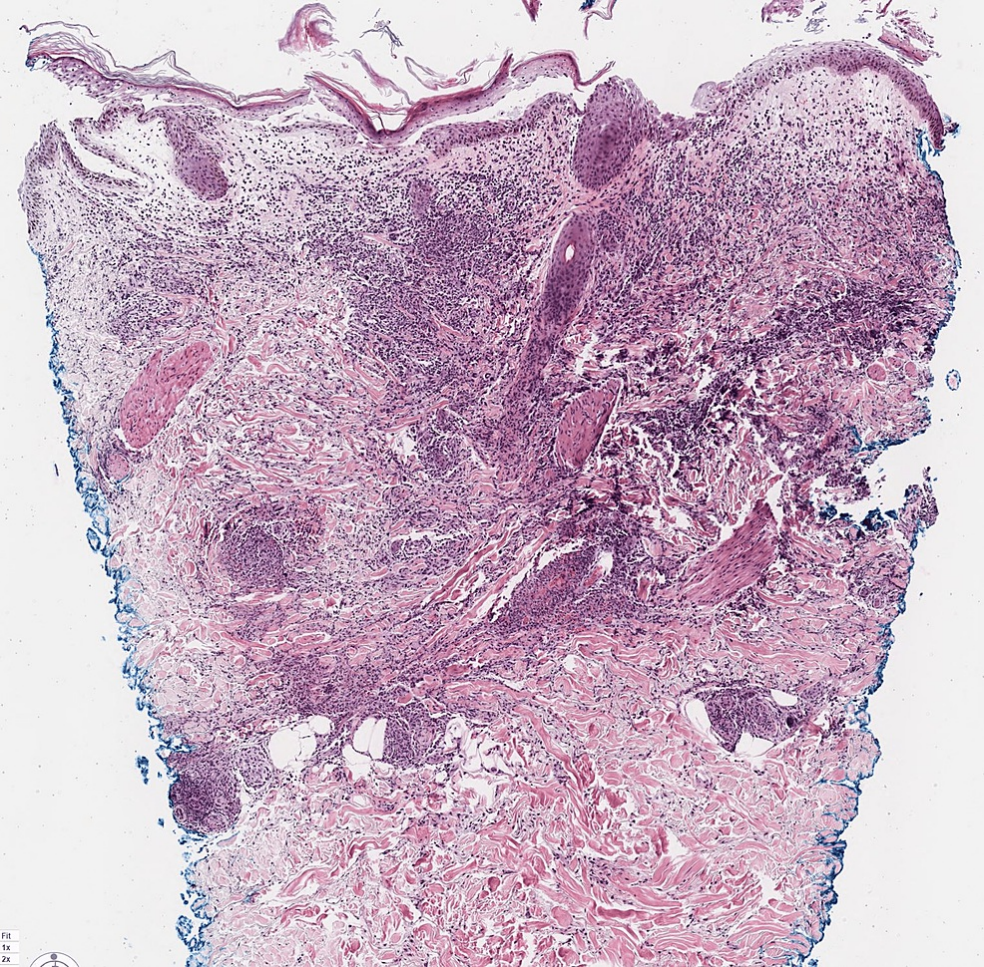
Punch biopsy specimen from lesion on the anterior thigh (H&E 4x). There is a dense interstitial and perivascular mixed inflammatory infiltrate involving the upper and mid-reticular dermis

**Figure 2:**
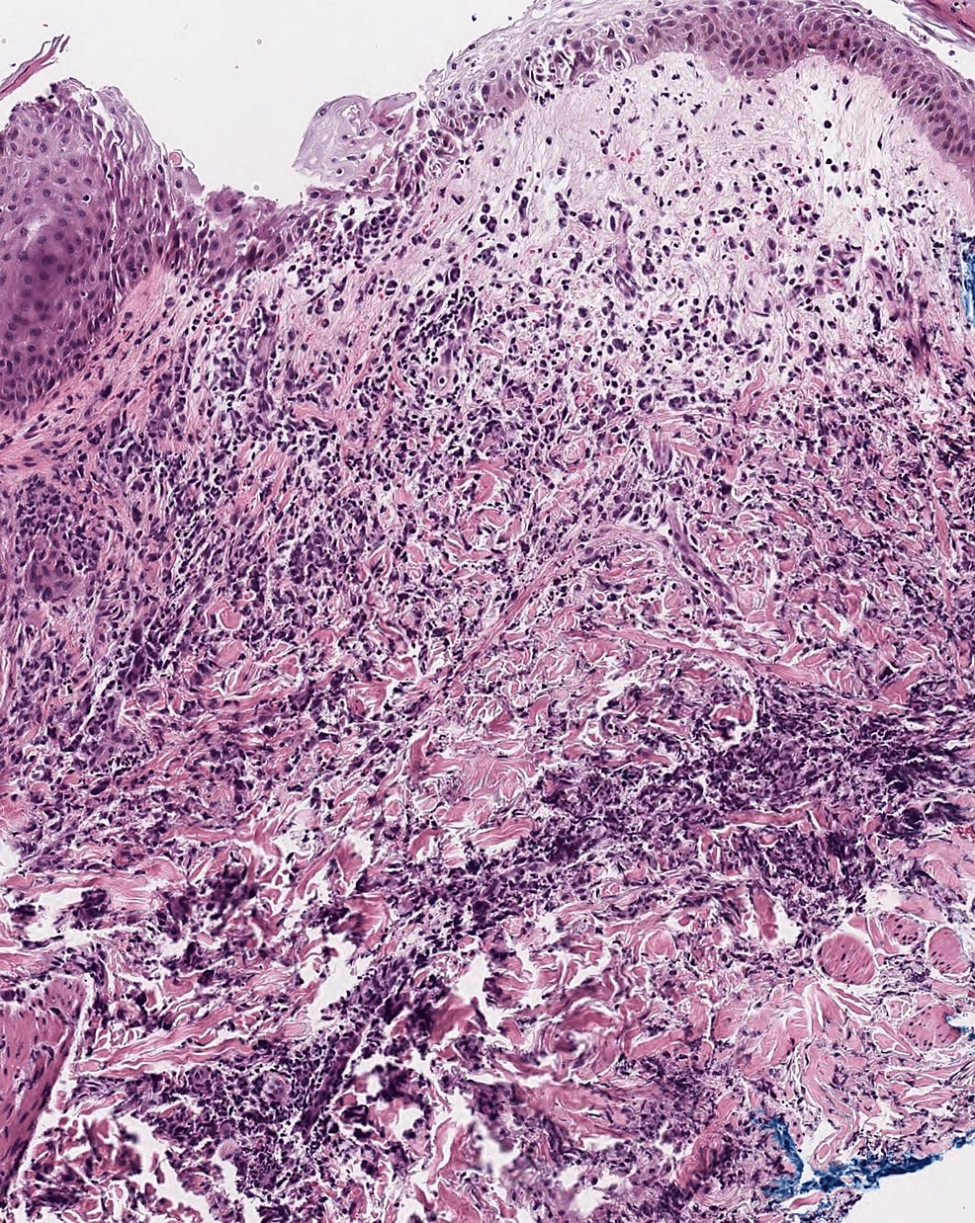
Punch biopsy specimen from lesion on the anerior thigh (H&E 10x). There is prominent papillary dermal edema with a pandermal mixed infiltrate that includes neutrophils demonstrating leukocytoclasis. Some of the neutrophils have histiocytoid morphology

**Figure 3:**
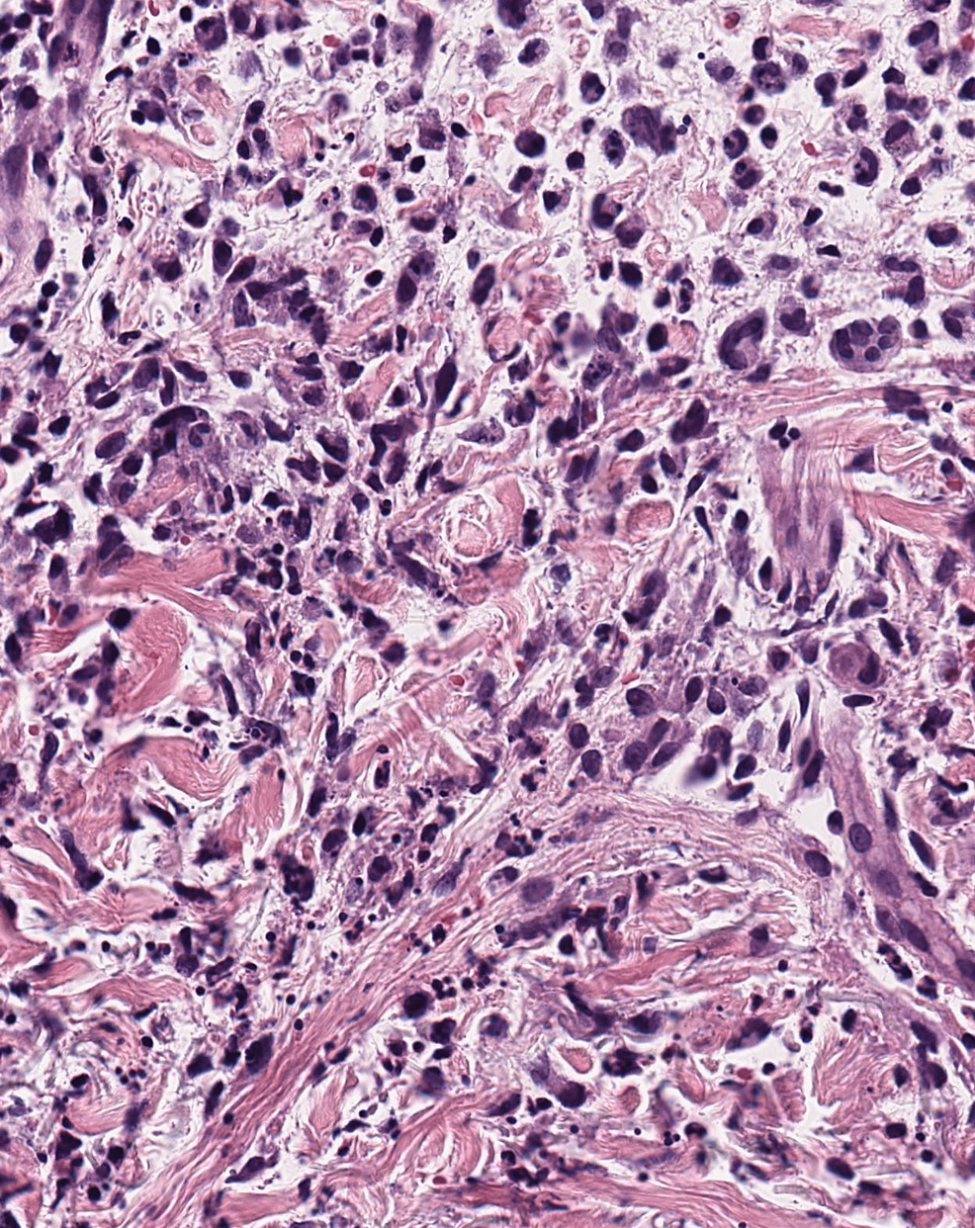
Higher power (H&E 40x) demonstrating an infiltrate of neutrophils with prominent leukocytoclasis

**Figure 4:**
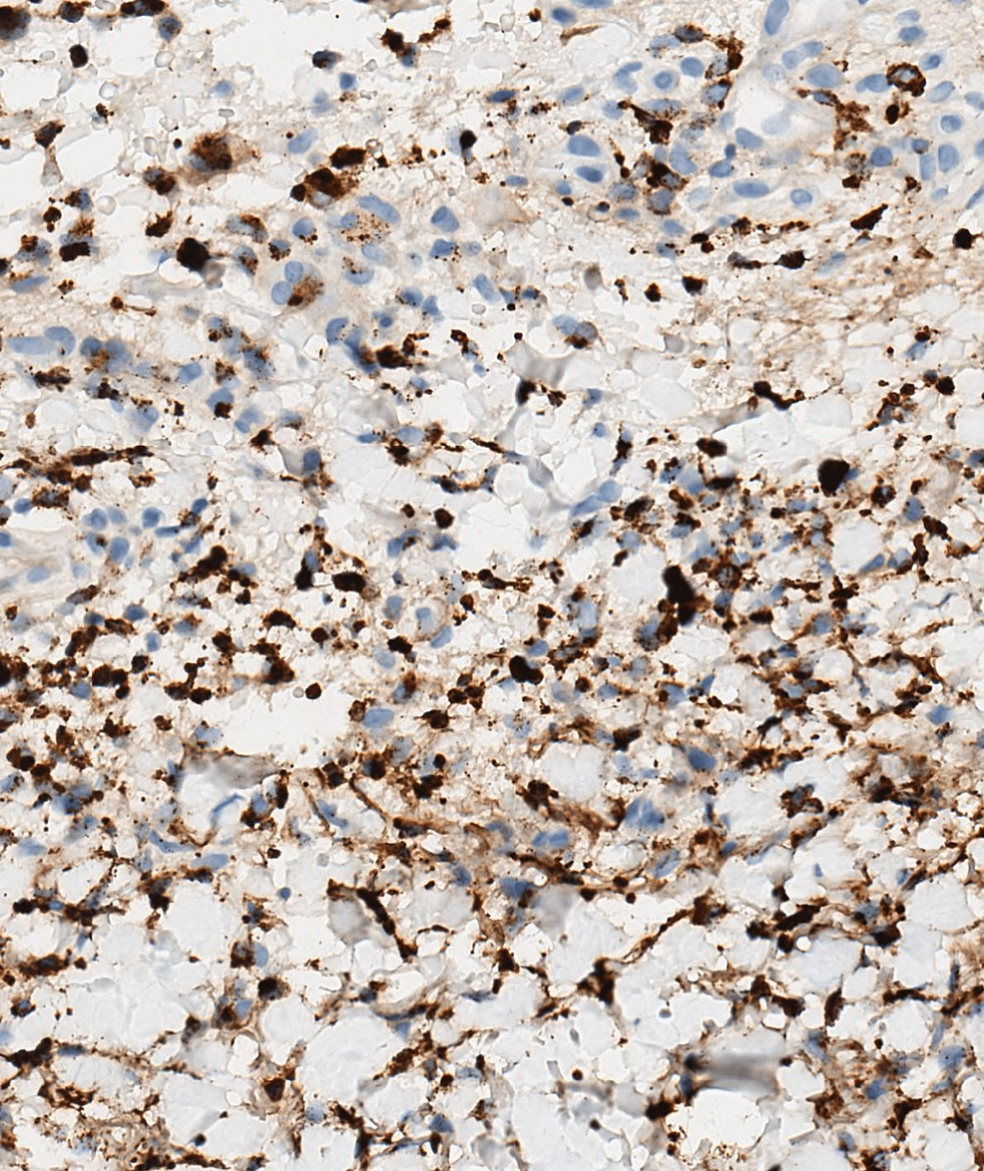
An immunohistochemical stain confirms the presence of neutrophils, positive for myeloperoxidase (Immunohistochemical stain for myeloperoxidase 40x)

A skin biopsy of her right anterior thigh lesion was performed with additional microbiological testing. Biopsy showed neutrophil-rich infiltrate leukocytoclastic and mild granulomatosis. There was prominent papillary dermal edema with a pan-dermal mixed infiltrate that includes neutrophils demonstrating leukocytoclasis. Some of the neutrophils had a histiocytoid morphology (Figures 1-3). There were areas of focal vascular disruption with intraluminal fibrin deposition consistent with focal vasculitis. An immunohistochemical stain confirmed the presence of neutrophils (Immunohistochemical stain for myeloperoxidase) (Figure 4). The lesions persisted as papules for a few days and, followed by some lesions progressing to pustules, which eventually crusted over. Lesions gradually and spontaneously resolved over the next few days in view of which steroids were deferred. The patient was discharged in stable condition with advice to follow up as an outpatient. 

## Discussion

We searched the Google Scholar and Pubmed databases for case reports, case series, and review articles on SS. We used the keywords “Sweet syndrome”, “malignancy”, “neutrophilic dermatosis”, “drug induced”, “review” and “infection”. Case reports, case series, and literature reviews from 1960 to 2022 were included. We compiled data from these sources to get insights into the etiology, epidemiology, pathophysiology, presentation, and histological features of SS. Commentaries, editorials, and articles not published in the English language were excluded. The included literature was reviewed by two independent reviewers for quality, power, and authenticity, and conflicts were resolved by discussion.

SS is an acute febrile neutrophilic dermatosis without vasculitis. This definition is by no means exclusive, and newer definitions and criteria have been proposed to account for atypical cases. Other diseases that fall into a similar category include, but are not limited to, pyoderma gangrenosum, pustular psoriasis, keratoderma blenorrhagicum, bowel associated dermatosis-arthritis syndrome, Behcets disease, acne fulminans, and familial Mediterranean fever.

Epidemiology

SS is a rare entity. The exact prevalence is unknown, likely owing to its rarity and stringent diagnostic criteria used thus far. Current prevalence estimates likely underestimate the actual burden of SS. Recent studies suggest the disease appears to affect all genders and races equally. Earlier studies reported different patterns of male-female distribution when SS was subclassified into classic, malignancy-associated, and drug-induced SS [3]. Classical SS is more common in adults and generally presents in the sixth decade of life unless it is associated with pregnancy [4]. Pediatric SS has also been described, with two cases described in young brothers at 10 and 15 days of life [5]. Age at presentation of malignancy-associated SS depends on the age at diagnosis of malignancy, and occurs with an equal frequency in males and females [6]. It can be present at the time of diagnosis of initial malignancy, or may precede the malignancy. According to some studies, about 21% of SS cases are associated with an underlying malignancy [7]. As noted previously, as per the currently accepted clinical criteria, a significant number of cases might be excluded at this point in time which makes any assessment of disease distribution poorly reliable. The actual patterns in the population can be different from those observed. 

Etiology

SS can present as a primary disease or secondary to malignancy, infections, autoimmune conditions, or drugs. Most authors in the past have classified SS into classic (idiopathic) SS, malignancy-associated SS, or drug-induced SS. In reality, the evidence based on the etiological basis of the disease is ever expanding with newer associations being proposed every year [8,3].

Classical SS is the most common and is usually associated with upper respiratory tract/gastrointestinal infections, inflammatory bowel disease (IBD), and pregnancy [3]. VEXAS (vacuoles, E1 enzyme, X-linked, autoinflammatory, somatic) syndrome has also been associated with classic SS. A relatively recent discovery, the first series of VEXAS syndrome was reported in 2020 in 25 men [9]. It is associated with multiple cutaneous manifestations. IBD-associated SS appears to be more common in middle-aged White females and occurs with relatively similar frequencies in both ulcerative colitis (UC) and Crohn’s disease (CD), although colonic involvement is especially common in CD-associated SS. In Asian populations, however, IBD-associated SS appears to be related to UC more than CD [10]. Azathioprine, one of the most effective and commonly used therapies in patients with IBD, is paradoxically one of the most common causes of drug-related SS. No criteria exist to differentiate between azathioprine and IBD related SS in patients with IBD who have SS, although the occurrence of azathioprine-induced IBD in older males might be a differentiating factor [10]. SS is also associated with inflammatory conditions. Inflammatory conditions described in the literature include systemic lupus erythematosus (SLE), rheumatoid arthritis, Sjogren’s syndrome, and IBD to name a few. 

Infections associated with classical SS include infections by Salmonella sp., Mycobacterium chelonae, and Penicillium sp. SS might be a presenting feature of coccidiomycosis and has also been linked to sporotrichosis, occurring more frequently with the S. brasiliensis subtype [11]. The list is virtually inexhaustible and includes various viral pathogens as well. More recently, several cases of SS have been reported with the SARS-COVID vaccine. Cases have been reported with Janssen Ad26.COV2.S vaccine, the Moderna mRNA01273 vaccine, inactivated SARS-CoV-2 vaccine ‘CoronaVac’, Oxford-AstraZeneca COVID-19 vaccine (AZD1222), and the BNT162b2 Pfizer/BioNTech vaccine. Few cases of Sweet syndrome have also been reported after influenza, Measles-Mumps-Rubella (MMR) and pneumococcal vaccinations.

SS has been reported in patients with myelodysplasia and acute promyelocytic leukemia [12]. Malignancy-associated SS has a temporal association with onsetof primary malignancy and affects men and women equally. It is associated with extracutaneous manifestations of SS in at least 50% of the cases including periorbital cellulitis, pyoderma gangrenosum, sudden massive swelling of the tongue in acute myeloid leukemia and erythematous tender plaques, and inflammatory changes in post-mastectomy lymphedema areas, which may simulate infection [6]. There also have been reports of cases of SS after receiving anti-cancer drugs including bortezomib, imatinib, lenalidomide, and all-trans retinoic acid (ATRA). SS appears to be more commonly associated with hematological, as opposed to solid organ, malignancies [13]. Hematological malignancy-associated SS frequently presents with cytopenias, requiring a high index of suspicion in these patients.

Several drugs are described in literature to have a possible association [14]. Granulocyte-colony stimulating factor (G-CSF), azathioprine, and ATRA are most commonly implicated in a growing list of medications. 

Pathophysiology

Pathophysiology is complex and multifactorial. It involves an interplay between neutrophil proliferation, decreased apoptosis, and cutaneous localization. Proliferation and reduced survival explain the association of the disease with hematological malignancies, G-CSF, and ATRA. Both ultraviolet (UV) light (through increased production of interleukin (IL)-8 and tumor necrosis factor-alpha (TNF-α) and the Koebner phenomenon (development of lesions at sites of skin trauma) have been postulated to contribute to cutaneous localization. Tumor cells in hematological malignancies contribute to cutaneous localization by producing TNF-α and IL-1B [15]. SS appears to have a poly-genetic basis, with the most plausible mechanism involving IDH1 mutations, inflammasome and IL-1B production [16]. High levels of IL-6 during active disease and lower levels with steroid treatment have been described in literature [16]. Patients with acute myelogenous leukemia (AML) who have SS have been shown to have a higher incidence of FLT3 mutations when compared to AML patients without SS; SS in AML does appear to be responsive to glucocorticoid therapy [17]. Patients with VEXAS syndrome have a missense mutation in codon 41 of *UBA1* gene, which encodes for the ubiquitin activating enzyme E1. These patients suffer from a spectrum of hematologic problems, including cytopenias, thromboembolic disease, and progressive bone marrow failure; all of which can evolve to a hematologic malignancy [9].

Presentation

SS classically presents with sudden onset of fever and flu-like symptoms followed by eruption of tender papules, plaques or nodules in an asymmetric distribution, classically over the face, neck, and extremities. Cutaneous lesions have a transparent, vesicle-like appearance attributable to pronounced edema in the upper dermis; some lesions morphologically resemble bullae. There is marked clinical variation in the characteristics and distribution of lesions [3]. Neutrophilic dermatosis of the hands is a distributional variant. Malignancy-associated SS may present as multiple different types of neutrophilic dermatoses in the same patient [18]. Fever may be absent in cases of malignancy-associated SS. Oral mucosa is also frequently involved [3]. Ocular involvement presents as conjunctivitis and/or episcleritis; uveitis, limbal nodules, glaucoma, subconjunctival hemorrhage, scleritis, iritis, and sudden visual loss have also been reported [19]. Extracutaneous involvement has been reported to involve multiple organ systems. Pulmonary involvement has been shown to respond well to corticosteroids [20]. Bone, intestine, joint, bone marrow, liver, heart, muscle, and spleen have also been reported in literature. Nervous system involvement presents as meningitis and/or encephalitis; peripheral neuropathy has also been reported. Neurological involvement, or the so-called “neuro Sweet disease”, was shown to have a strong human leukocyte antigen (HLA) Cw-1 association in a study on Japanese population, and it did respond to corticosteroids, although recurrences were common [21]. Neurological manifestations can precede cutaneous disease by several years. Absence of arthralgias is also likely associated with increased risk of malignancy [4]. Photosensitive SS can mimic cutaneous SLE but has different histological features and negative immunofluorescence [22]. SS associated with adult-onset immunodeficiency tends to have pustular lesions, leukocytosis, and lymphadenopathy [23]. 

Over time, three clinical variants of SS have been described, based on appearance of the skin lesions. Of note, different types of lesions may be encountered in the same patient; this classification lacks support from established guidelines. Bullous SS presents with tense or flaccid bullae or blisters. Histology shows separation of epidermis and dermis from edema of the papillary dermis. Bullae may be present in up to 30% of cases of SS. Giant cellulitis-like SS (GCL-SS) presents with lesions similar to those of cellulitis or erysipelas unresponsive to initial antibiotic therapy and with negative blood cultures; high index of suspicion is required to avoid invasive procedures for management of presumed antibiotic resistant cellulitis [24]. Diagnosis of GCL-SS is supported by the simultaneous appearance of multiple lesions in the absence of lymphadenopathy. The first three cases were reported in 2013; all three patients were obese with BMIs of more than 35 [24]. Necrotizing SS was first described in 2012 by Kroshinsky et al. Since then, more cases have come to clinical attention. It presents similarly to rapidly progressive necrotizing fasciitis unresponsive to antibiotics. Like GCL-SS, this also presents a diagnostic challenge as surgical management for presumed necrotizing fasciitis can lead to exacerbation of true necrotizing SS. 

Histology

Several histological subtypes of SS exist. Histiocytoid SS (HSS) was first described in 2005 in a study of 41 patients with clinical features of SS and a dermal infiltrate composed mostly of mononuclear cells. HSS affects males and females equally and is more likely to be associated with an underlying malignancy than classical SS [25]. Lesions in HSS demonstrate histiocytic mononuclear appearing cells that are differentiated from immature myeloid cells with the help of immunohistochemical stains, although this is difficult since very few immunohistochemical stains are specific to histiocytes and immature myelocytes [26]. Studies have demonstrated myeloperoxidase in both early and late lesions of SS, suggesting that early lesions may have immature, histiocyte appearing, immature myeloid cells, with mature neutrophils appearing in later stages [26]. Cutaneous involvement by RAS-associated autoimmune leukoproliferative disease (RALD) may present as HSS, and usually occurs in the setting of RALD associated with RAS mutations [27]. Immunohistochemical and histopathologic analysis of samples from 33 cases reported the inflammatory infiltrate in HSS to be mainly composed of myeloperoxidase-positive immature myelomonocytic cells with histiocytoid morphology and provided a basis for differentiation between malignancy associated SS and leukemia cutis [28].

Cryptococcoid SS (CSS) is a very rare entity, first described in 2013 although the terminology itself was coined in 2017 [29]. In CSS, skin biopsy shows dermal inflammatory infiltrate with neutrophils with bilobed nuclei surrounded by vacuolated spaces which coalesce to form a perinuclear halo. Negative fungal stains exclude cryptococcosis and immunohistochemistry stains positively for myeloperoxidase [30]. Moreover, these patients fail to respond to antifungal treatment but do seem to respond to corticosteroids [31]. The etiology for development of these histological features is unclear; researchers have postulated lab artifacts and neutrophil apoptosis as the possible underlying pathological processes [31]. 

Subcutaneous SS (SSS) involves the subcutaneous tissues and presents with neutrophilic lobular panniculitis with or without involvement of septae in the subcutaneous fat [32]. Mild fat necrosis may be present. Neutrophilic panniculitis is a non-specific histological finding and is seen in patients with Behcet's disease, erythema nodosum, pyoderma gangrenosum and erythema multiforme, among others. The necrotizing variant mimics necrotizing fasciitis and involves deeper tissues such as muscle and associated fascia. It is differentiated from necrotizing fasciitis by the absence of infection. Again, differentiation is important to avoid debridement. SSS is a diagnosis of exclusion. 

Histological findings in patients with VEXAS syndrome-associated SS include perivascular infiltrate extending into the dermis and even the subcutaneous at in some cases. Dermal infiltrates in VEXAS syndrome are derived from the same pathological *UBA1*-mutated myeloid clone found in the bone marrow [33].

Although the criteria proposed by Von Den Driesch excludes vasculitis, fibrinoid necrosis of the vessel walls with neutrophilic infiltration has been demonstrated in various biopsy specimens taken from patients originally diagnosed with SS [34]. Negative immunofluorescence is suggestive of vasculitis in SS being a result of noxious products released from neutrophils and not due to immune complex deposition.

Diagnostic Criteria

The diagnostic criteria for classical SS was first proposed by Su and Liu in 1986 [35]. This was subsequently revised by Von Den Driesch [34]. Criteria for drug-induced SS was proposed by Walker and Cohen [36]. Diagnostic criteria for malignancy-associated SS is similar to that of classic SS, except for the substitution of “underlying malignancy” as a minor criterion in place of “inflammatory disease, pregnancy, vaccination, or infection” [34]. Again, the requirement for absence of vasculitis makes the diagnosis questionable in many cases. A more recently proposed set of criteria for SS focuses on constant and variable features in place of major and minor criteria. It suggests that two constant features are sufficient to diagnose SS irrespective of variable features [37]. The newer criteria allows for the inclusion of vasculitis, potentially allowing for early initiation of treatment while avoiding further, often unnecessary, diagnostic testing. This was the case in our patient, who fulfilled two minor criteria (leukocytosis, elevated ESR, and CRP) and the two major criteria with the exception of vasculitis.

Management

Although response to steroids is a component of the diagnostic criteria, no established guidelines exist for management of SS. Oral, topical, and intralesional steroid formulations have all been used with satisfactory results. Second line agents include indomethacin, clofazimine, cyclosporine, and dapsone [3]. Potassium iodide inhibits neutrophil chemotaxis making it a suitable option in SS [38]. Colchicine has also been shown to be effective [3]. Treatment with TNF-α and IL-1B inhibitors has been tested with promising results [39]. More recently, granulocyte and monocyte adsorption apheresis (GMA) has gained popularity in the treatment of SS and is generally well tolerated [40]. Biological agents can be a useful second-line option in patients with IBD-associated SS [10]. Most of this information is available from case reports; absence of clinical trials to study effective and safe treatment options is a gap in the existing literature. Of note, in our patient, the lesions resolved spontaneously.

## Conclusions

SS is a diverse combination of clinicopathologic and histological observations that occurs in association with or secondary to an even more diverse range of etiological factors. Features that remain consistent are the presence of neutrophilic inflammation in the absence of underlying infection or immune complex deposition, and a prompt response to steroids. The evidence base on SS has grown and evolved. This has led to newer diagnostic models that attempt to include atypical presentations. In light of the existing evidence, we agree to the proposed revision of including atypical cases by excluding vasculitis as a required feature, since this requirement may well have led to underreporting in the past. At this time, corticosteroids continue to be the mainstay of treatment, although newer treatment strategies are evolving and are proving to be safe and efficacious. Early and accurate histological diagnosis is of paramount significance not only to start a relatively safe and effective treatment in a timely manner but also to start the workup of a potentially serious underlying diagnosis.
